# Predictive Ability of Serum IL-27 Level for Assessing Activity of Antineutrophil Cytoplasmic Antibody-Associated Vasculitis

**DOI:** 10.1155/2021/6668884

**Published:** 2021-07-19

**Authors:** Taejun Yoon, Sung Soo Ahn, Jung Yoo Pyo, Lucy Eunju Lee, Jason Jungsik Song, Yong-Beom Park, Sang-Won Lee

**Affiliations:** ^1^Department of Medical Science, BK21 Plus Project, Yonsei University College of Medicine, Seoul, Republic of Korea; ^2^Division of Rheumatology, Department of Internal Medicine, Yonsei University College of Medicine, Seoul, Republic of Korea; ^3^Institute for Immunology and Immunological Diseases, Yonsei University College of Medicine, Seoul, Republic of Korea

## Abstract

Serum interleukin- (IL-) 27 level has been reported to increase in patients with several autoimmune diseases; however, its significance in patients with antineutrophil cytoplasmic antibody- (ANCA-) associated vasculitis (AAV) is unknown. In this study, we investigated the associations between serum IL-27, laboratory features, and activity of AAV and evaluate the predictive ability of serum IL-27 level for disease activity. This study included 77 AAV patients, and we collected clinical and laboratory data at blood sampling. Inflammation-related variables included white blood cell, neutrophil, lymphocyte and platelet counts, serum albumin, erythrocyte sedimentation rate, and C-reactive protein levels. Serum IL-27 and IL-18 levels were measured from stored sera using Human Magnetic Luminex® assay. High disease activity of AAV was defined as the highest tertile of Birmingham vasculitis activity score (BVAS) (≥11). The mean age of the enrolled patients was 59.9 years, and 38 (49.4%) were diagnosed as microscopic polyangiitis. In the multivariable analysis, serum albumin (*β* = −0.419) and serum IL-27 level (*β* = 0.221) were significantly associated with BVAS. Furthermore, patients with renal manifestation exhibited higher serum IL-27 (mean 308.7 pg/mL vs. 105.8 pg/mL) and IL-18 levels (mean 376.7 pg/mL vs. 270.4 pg/mL) than those without. On applying the optimal cut-off of serum IL-27 level for predicting high activity, AAV patients with serum IL − 27 level ≥ 300.8 pg/mL had a significantly higher risk for having high disease activity than those with serum IL − 27 level < 300.8 pg/mL (relative risk 3.380, 95% confidence interval 1.223, 9.345, *P* = 0.016). These results suggest that serum IL-27 level is associated with the cross-sectional activity and the presence of renal manifestation and could be used to predict high disease activity in patients with AAV.

## 1. Introduction

Interleukin- (IL-) 27 is a member of the IL-6/IL-12 family of cytokines mainly produced by antigen-presenting cells such as dendritic cells, monocytes, and macrophages and is consisted of two subunits, Epstein-Barr virus-induced gene 3 and p28 [1]. The role of IL-27 in mediating inflammatory and autoimmune processes is still controversial but largely considered to be proinflammatory [2]. Importantly, IL-27 induces the proliferation of T and B cells, which can drive the inflammatory response through enhancement of acquired immunity [1, 3]. With regard to T follicular helper (TFH) cells, IL-27 improves its function and increases the production of IL-21, an autocrine cytokine that maintains and stabilises the function of TFH cells. In addition, activated TFH cells promote the production of immunoglobulins by B cells and accelerate class switching of immunoglobulins closely related to inflammatory or autoimmune diseases [4]. Meanwhile, IL-27 stimulates the differentiation of TH17 cells and develops their function by augmenting the production of IL-23, IL-17, IL-6, and IL-1*β* to sustain the altered immune response [5, 6]. Accordingly, previous studies that investigated serum IL-27 levels in autoimmune diseases reported that serum IL-27 is increased in patients with rheumatoid arthritis, systemic lupus erythematosus, and systemic sclerosis compared to those in healthy subjects [7–9].

Currently, systemic vasculitides are classified according to the size of the invading vessels and the pathologic findings. Among them, small vascular vasculitis (SVV) is divided into immune complex SVV and antineutrophil cytoplasmic antibody- (ANCA-) associated vasculitis (AAV) based on the presence of immune complex deposits. AAV primarily involves the capillaries, arterioles, and venules and occasionally affects arteries, which shows a critical histological feature of necrotising vasculitis with few or no immune complex deposits [10]. AAV is differentiated into three subtypes including microscopic polyangiitis (MPA), granulomatosis with polyangiitis (GPA), and eosinophilic granulomatosis with polyangiitis (EGPA) according to clinical features, laboratory results, and histological findings [10, 11]. While it has been reported that AAV is a rare disorder with an estimated incidence and prevalence of 1.2-3.3/100,000 and 4.6-42.1/100,000 [12], recent studies indicate that its occurrence is rising owing to the advances in the understanding of disease and improvements in diagnostic tests. Of note, given that it is associated with increased risk of mortality and morbidity especially in the presence of increased disease activity, persistent interests have been present in identifying laboratory markers that could aid in assessing the disease status of AAV.

Although it has not been described in the literature whether IL-27 is directly linked with the pathophysiology of AAV, there is an increasing body of evidence suggesting that IL-27 may be associated with heightened inflammation in AAV. First, in the pathogenesis of AAV, IL-23 and IL-17 are cytokines that could prime neutrophils, as well as initiate and amplify the production of ANCA by B cells. Furthermore, they activate immune cells including T cells, B cells, and macrophages and accelerate the complement pathway, resulting in vascular damage [13, 14]. On the other hand, circulating TFH cells were reported to be significantly increased in AAV or GPA patients compared to healthy controls [15, 16]. Furthermore, circulating IL-21, which plays an important role in activating TFH cells, was also reported to be higher in AAV or GPA patients than in healthy controls [17], and we previously demonstrated that serum IL-21-positive AAV patients exhibited a higher cross-sectional Birmingham vasculitis activity score (BVAS) than serum IL-21-negative AAV patients [18].

Taking into consideration that IL-27 is considered to be an upstream regulator that could influence in the differentiation and function of TFH, TH17, and B cells, it may be possible that serum IL-27 level may be relevant to the disease severity of AAV. Of note, a previous study by Shen et al. demonstrated that serum IL-27 level was increased in Behçet's disease—which is a variable vessel vasculitis—with active ocular inflammation [19]. However, to the best of our knowledge, no study has been performed to investigate the significance of serum IL-27 level in patients with AAV. Hence, the aim of the present study was to investigate (i) whether there are associations between serum IL-27, laboratory features, and activity and (ii) evaluate the predictive ability of serum IL-27 level for the disease activity of AAV.

## 2. Materials and Methods

### 2.1. Patient Selection and Severance Hospital ANCA-Associated VasculitidEs (SHAVE) Cohort

This study was performed by using the blood samples and data of 77 patients who were enrolled in the SHAVE cohort. The SHAVE cohort is a prospective observational cohort in Severance Hospital of patients with MPA, GPA, and EGPA: patients were classified as disease subtypes of AAV according to the 2007 European Medicines Agency (EMA) algorithm and the 2012 revised International Chapel Hill Consensus Conference Nomenclature of Vasculitides [10, 11]. In our cohort, whole blood is drawn after obtaining patient consent and sera is immediately isolated from whole blood and stored at -80°C. On the same visit day, routine inflammation-related laboratory tests and AAV-specific indices of BVAS (version 3) and five-factor score (FFS) are also assessed to evaluate disease activity and prognosis [20, 21]. Furthermore, blood collection and assessment of laboratory and clinical data are regularly performed on every three to six months' interval. As indicated in the entry requirement of the 2007 EMA algorithm, we excluded subjects with concomitant serious infections, malignancies, or secondary vasculitis other than AAV, which were identified using the 10th revised International Classification Diseases. This study was approved by the Institutional Review Board (IRB) of Severance Hospital (4-2016-0901), and written informed consent was obtained from all of the patients.

### 2.2. Clinical and Laboratory Data

All clinical and laboratory data were used at the period when the collection of sera was done. Age and sex were included as demographic data, and disease duration, AAV subtype, and ANCA positivity at the time of blood sampling were reviewed. Of 77 patients, 40 (51.9%) provided whole blood within one month of AAV diagnosis and were considered newly diagnosed AAV. In addition, the presence of clinical manifestations was evaluated based on the subcategories comprising BVAS version 3 [20]. Inflammation-related variables consisted of the white blood cell (WBC), neutrophil, lymphocyte and platelet counts, serum albumin, erythrocyte sedimentation rate (ESR), and C-reactive protein (CRP) levels. Immunosuppressive drugs that the patients are currently on prescription were identified using the Korean Drug Utilisation Review system. Serum IL-27 and IL-18 levels in stored sera were measured using the Human Magnetic Luminex® assay (R&D Systems, USA) according to the manufacturer's instructions.

### 2.3. Measurement of ANCA Positivity

ANCA positivity was interpreted according to the revised 2017 international consensus on testing of ANCAs in GPA and MPA [22]. We used immunoassays as the primary screening method for ANCA. Myeloperoxidase- (MPO-) ANCA and proteinase 3- (PR3-) ANCA were measured using the novel anchor-coated highly sensitive Phadia ELiA (Thermo Fisher Scientific/Phadia, Freiburg, Germany) and the Phadia250 analyser. However, when patients were found to be negative for ANCA by an antigen-specific assay but positive by an indirect immunofluorescence assay, they were also considered to be MPO-ANCA or PR3-ANCA positive based on the discretion of treating physician [23].

### 2.4. Statistical Analyses

Statistical analyses were performed using the SPSS software (version 25 for Windows; IBM Corp., Armonk, NY, USA). Continuous variables are expressed as mean ± standard deviation, whereas categorical variables are expressed as numbers (percentages). The correlation coefficient (*r*) between the two variables was obtained by the Pearson correlation analysis. The standardised correlation coefficient (*β*) was obtained by the multivariable linear regression analysis using variables with statistical significance in the univariable analysis. Significant differences between continuous variables were determined using the Student *t*-test, while the chi-square test or Fisher's exact test was carried out for the comparison of categorical variables. Patients were stratified into three groups based on the tertile of BVAS, and we defined the lower limit of the highest tertile as the cut-off for high activity of AAV (BVAS ≥ 11). The optimal cut-off of serum IL-27 level for high activity of AAV was determined using the receiver operator characteristic (ROC) curve analysis. The relative risk (RR) of the cut-off of serum IL-27 for high activity of AAV was analysed using contingency tables and the chi-square test. Two-tailed *P* values of less than 0.05 were considered statistically significant.

## 3. Results

### 3.1. Clinical Characteristics of Patients

The mean age and the disease duration of the patients enrolled were 59.9 years and 18.2 months, respectively. Among the 77 patients, 44 (57.1%) and 33 (42.9%) of the patients were female and male, respectively. The diagnosis of MPA (49.4%), followed by GPA (31.2%), and EGPA (19.5%), was the most common subtypes of AAV. In addition, ANCA positivity was found in 55 of the 77 patients, and the mean BVAS and FFS were assessed as 9.8 and 1.3. Clinical manifestation of pulmonary manifestation (58.4%) and renal manifestation (53.2%) were the most common in the patients. The mean ESR and CRP were measured as 42.9 mm/h and 20.0 mg/L, and the mean levels of serum IL-27 and IL-18 were 213.7 pg/mL and 327.0 pg/mL, respectively (Table 1). When the patients were divided according to sex, renal manifestation was found to be more frequent and serum IL-18 level was higher in male; however, no other difference was noted between the other variables investigated.

### 3.2. Linear Regression Analysis with Cross-Sectional BVAS

A linear regression analysis was performed to evaluate the relationship between inflammation-related variables, IL-27, IL-18, and BVAS. In the univariable linear regression analysis, all variables except for lymphocyte count and serum IL-18 level were significantly correlated with the cross-sectional BVAS. In the multivariable analysis, both serum albumin (*β* = −0.419; 95% confidence interval (CI) -7.410, -1.635; *P* = 0.003) and serum IL-27 level (*β* =0.221; 95% CI 0.001, 0.015; *P* = 0.025) were revealed to be related to BVAS (Table 2).

### 3.3. Relationship between Inflammation-Related Variables and Serum IL-18 and Serum IL-27 Levels

Among inflammation-related variables and serum IL-18 level, only serum albumin (*r* = −0.384, *P* = 0.001) and serum IL-18 level (*r* = 0.478, *P* < 0.001) were significantly correlated with serum IL-27 level (Table 3).

### 3.4. Comparison of Serum IL-27 and IL-18 Levels Based on the Presence and Absence of Clinical Manifestations

Upon comparing serum IL-27 and IL-18 levels according to the presence of each clinical manifestation, only renal manifestations significantly differed. Serum IL-27 (mean 308.7 pg/mL vs. 105.8 pg/mL, *P* < 0.001) and IL-18 (mean 376.7 pg/mL vs. 270.4 pg/mL, *P* = 0.003) levels in patients with renal manifestation were significantly higher than those in patients without renal manifestation (Table 4).

### 3.5. The Optimal Cut-Off of Serum IL-27 Level for High Activity of AAV

**Figure 1:**
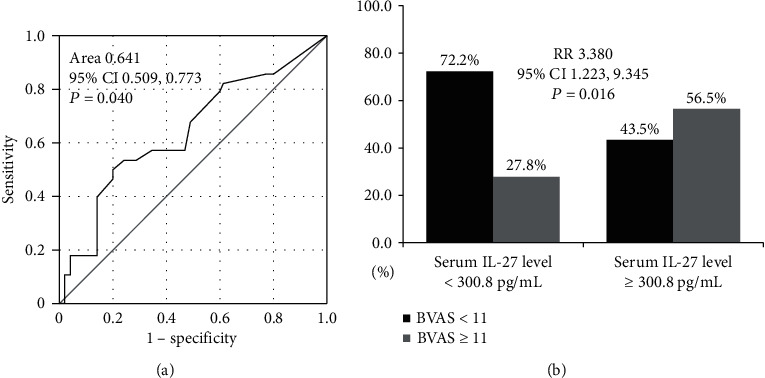
Cut-off of serum IL-27 level for high activity of AAV and relative risk. (a) The optimal cut-off of the serum IL-27 level for predicting high activity of AAV was set as 300.8 pg/mL. (b) AAV patients with serum IL-27 level ≥ 300.8 pg/mL had a significantly higher risk of having high activity than those without. IL: interleukin; ANCA: antineutrophil cytoplasmic antibody; AAV: ANCA-associated vasculitis; RR: relative risk.

Using the ROC curve, the optimal cut-off of serum IL-27 level for estimating high activity of AAV was set as 300.8 pg/mL, with a sensitivity and specificity of 50.0% and 79.6% (area 0.641; 95% CI 0.509, 0.773; *P* = 0.040) (Figure 1(a)).

Next, when AAV patients were divided into two groups based on the serum IL-27 level of 300.8 pg/mL, 23 of 77 (29.9%) patients were categorized into the group with serum IL-27 level ≥ 300.8 pg/mL. High activity of AAV was identified more frequently in AAV patients with serum IL-27 level ≥ 300.8 pg/mL than those with serum IL-27 level < 300.8 pg/mL (56.5% vs. 27.8%, *P* = 0.016). Moreover, the risk of having high disease activity was significantly higher in patients with serum IL-27 level ≥ 300.8 pg/mL compared to those without (RR 3.380, 95% CI 1.225, 9.345) (Figure 1(b)).

## 4. Discussion

In this study, we investigated the predictive ability of serum IL-27 level for the activity of AAV and four novel findings were found. First, serum IL-27 level was significantly associated with BVAS and independently predicted the cross-sectional activity of AAV. Second, serum IL-27 level was negatively correlated with serum albumin and positively correlated with serum IL-18 level. Third, patients with renal manifestation exhibited higher serum IL-27 and IL-18 levels than those without. Fourth, AAV patients with serum IL-27 level ≥ 300.8 pg/mL had a significantly higher risk for high activity of AAV than those with serum IL-27 *level* < 300.8 pg/mL (RR 3.380). Therefore, we suggest that serum IL-27 level may be a useful biomarker for predicting the cross-sectional activity of AAV.

Among the versatile function of IL-27 in influencing the adaptive immune system [24], two different functions of IL-27 may be taken into account for explaining the dysregulated immune response in AAV. First, IL-27 enhances the production of immunoglobulin in B cells by stimulating and activating TFH cells in the germinal centre [4]. Second, IL-27 is involved in the production of cytokines responsible for priming neutrophils; thus, increased IL-27 may indirectly facilitate the binding of autoantigens on the cell surface of neutrophils and circulating ANCAs. Subsequently, neutrophils activated by ANCAs could initiate vascular inflammation, and autoreactive B cells are responsible for the sustained ANCA production [13, 14]. For these reasons, we hypothesized that serum IL-27 level may have a positive impact in perpetuating inflammation in AAV. When we compared serum IL-27 levels between patients with and without MPO-ANCA (or P-ANCA) and PR3-ANCA (C-ANCA), it was found that patients with MPO-ANCA (or P-ANCA) exhibited a significantly higher serum IL-27 level than those who did not (mean 249.9 pg/mL vs. 148.8 pg/mL, *P* = 0.037). However, the presence of PR3-ANCA (or C-ANCA) did not affect serum IL-27 level, which may in part, suggest that IL-27 could contribute to the inflammation in AAV through both the ANCA-dependent and ANCA-independent pathways.

In this study, we analysed IL-18 level as well as IL-27, because IL-27 has also shown to regulate immune response via IL-18 and its binding protein (BP) [25, 26]. Our results demonstrated that serum IL-27 level was highly correlated with serum IL-18 level; however, serum IL-18 level did not correlate with BVAS different from serum IL-27 level. Although there was a discrepancy between predictions of the cross-sectional activity of AAV using serum IL-27 level and serum IL-18 level, both serum IL-27 and serum IL-18 levels were significantly associated with renal manifestation (Table 4). Accordingly, we also analysed the correlation of serum IL-27 level and serum IL-18 level with the total score of each clinical item of BVAS. Serum IL-27 and IL-18 levels were significantly correlated with only the total score of renal manifestation among the nine items (*r* = 0.514, *P* < 0.001 and *r* = 0.256, *P* = 0.025, respectively) (Table 5). In contrast to IL-18BP, which is an inhibitor of IL-18 activity, IL-18 is reported to strengthen neutrophil priming and accelerates the renal progression of AAV [27, 28]. Therefore, IL-18 could be associated with renal involvement of AAV although IL-18 could not independently predict the cross-sectional activity of AAV.

Since BVAS is a composite measure comprising a wide range of clinical, laboratory, and radiological items, it has been considered the most reliable tool to predict the cross-sectional activity of AAV [20, 29]. Nevertheless, in actual clinical practice, a substantial burden of the time is required to accurately measure many items consisting BVAS. Therefore, the discovery of a biomarker to predict the cross-sectional activity of AAV in place of BVAS is expected to have high clinical utility. Our results indicate that measuring serum IL-27 level could have clinical implications in the management of AAV. Notably, the clinical efficacy of serum IL-27 levels in predicting the cross-sectional activity of AAV was further investigated by dividing patients into two groups according to the diagnosis time to exclude the influence of disease duration in evaluating the relationship between IL-27 and BVAS. In 40 patients with newly (within one month) diagnosed AAV, serum albumin (*r* = −0.488), serum IL-27 level (*r* = 0.420), and serum IL-18 level (*r* = 0.431) were significantly correlated with the cross-sectional BVAS. Meanwhile, in 37 patients with previously diagnosed AAV at the time of blood sampling, WBC count (*r* = 0.354), neutrophil count (*r* = 0.472), and serum IL-27 level (*r* = 0.367) were significantly correlated with the cross-sectional BVAS (Table 6). The results were different between the two groups, but only serum IL-27 level consistently showed a significant correlation with the cross-sectional activity of AAV. This suggests that serum IL-27 level could predict the cross-sectional BVAS regardless of the time of diagnosis when performing the test. Since serum IL-18 level showed a significant correlation with the cross-sectional BVAS only in newly diagnosed patients, it is expected that it can be used as a biomarker to predict BVAS at diagnosis, which should be investigated in depth by future studies.

The advantage of our study is that this study demonstrated for the first time that serum IL-27 level could predict the cross-sectional activity of AAV, and in particular, it proved its role as a biomarker regardless of the time of diagnosis. However, we acknowledge that our study possesses several limitations. This study included a relatively small number of patients as it was a single-centre study and did not include healthy controls or a validation cohort, which may reduce its clinical significance. However, we believe that it has clinical implications in that it is the first to discover the predictive ability of serum IL-27 level for the cross-sectional activity of AAV as a pilot study. In the near future, studies that include a larger number of patients and controls are especially warranted providing reliable evidence regarding the clinical relevance of serum IL-27 level in patients with AAV.

## 5. Conclusions

In conclusion, serum IL-27 level was associated with the cross-sectional activity of AAV and the presence of renal manifestation and was predictive of high disease activity in patients with AAV, indicating that it could have clinical implications in patients with AAV.

## Figures and Tables

**Table 1:** Characteristics of 77 patients with AAV.

Variables	Total (*n* = 77)	Female (*n* = 44)	Male (*n* = 33)	*P* value
Demographic data				
Age (years)	59.9 (14.5)	59.7 (14.5)	60.1 (14.7)	0.891
Disease duration (months)	18.2 (37.5)	17.1 (30.3)	19.6 (45.9)	0.785
AAV subtypes (*N*, (%))				
MPA	38 (49.4)	22 (50.0)	16 (48.5)	0.896
GPA	24 (31.2)	12 (27.3)	12 (36.4)	0.397
EGPA	15 (19.5)	10 (22.7)	5 (15.2)	0.409
ANCA positivity (*N*, (%))				
MPO-ANCA (or P-ANCA) positive	49 (63.6)	29 (65.9)	20 (60.6)	0.634
PR3-ANCA (or C-ANCA) positive	9 (11.7)	6 (13.6)	3 (9.1)	0.725
Both ANCA positive	3 (3.9)	2 (4.5)	1 (3.0)	0.999
ANCA negative	22 (28.6)	11 (25.0)	11 (33.3)	0.426
AAV-specific indices				
BVAS	9.8 (7.6)	8.9 (7.4)	11.0 (7.8)	0.228
FFS	1.3 (0.9)	1.2 (0.9)	1.5 (0.9)	0.161
Clinical manifestations (*N*, (%))				
General manifestation	28 (36.4)	17 (38.6)	11 (33.)	0.634
Cutaneous manifestation	7 (9.1)	4 (9.1)	3 (9.1)	0.999
Mucous and ocular manifestation	3 (3.9)	3 (6.8)	0 (0.0)	0.256
Otorhinolaryngologic manifestation	34 (44.2)	23 (52.3)	11 (33.3)	0.100
Pulmonary manifestation	45 (58.4)	24 (54.5)	21 (63.6)	0.426
Cardiovascular manifestation	4 (5.2)	2 (4.5)	2 (6.1)	0.999
Gastrointestinal manifestation	1 (1.3)	1 (2.3)	0 (0.0)	0.999
Renal manifestation	41 (53.2)	18 (40.9)	23 (69.7)	0.013
Nervous systemic manifestation	21 (27.3)	14 (31.8)	7 (21.2)	0.304
Inflammation-related variables				
WBC count (/mm^3^)	8,646.8 (4,285.0)	8,380.5 (4,602.8)	9,001.8 (3,861.7)	0.532
Neutrophil count (/mm^3^)	6,177.9 (3,424.8)	5,929.5 (3,485.0)	6,509.1 (3,367.2)	0.466
Lymphocyte count (/mm^3^)	1,503.6 (763.0)	1,442.5 (717.0)	1,585.2 (824.4)	0.421
PLT count (×1,000/mm^3^)	303.5 (144.4)	293.9 (147.8)	316.4 (140.9)	0.502
Serum albumin (mg/dL)	3.7 (0.7)	3.8 (0.6)	3.6 (0.8)	0.180
ESR (mm/h)	42.9 (36.3)	44.0 (41.0)	41.5 (29.4)	0.763
CRP (mg/L)	20.0 (40.5)	21.6 (42.7)	17.9 (37.9)	0.697
Serum cytokines				
IL-27 (pg/mL)	213.7 (214.7)	193.2 (217.2)	241.3 (211.3)	0.333
IL-18 (pg/mL)	327.0 (160.8)	291.0 (143.5)	375.1 (172.0)	0.022

Values are expressed as a mean ± standard deviation or *N* (%). ANCA: antineutrophil cytoplasmic antibody; AAV: ANCA-associated vasculitis; MPA: microscopic polyangiitis; GPA: granulomatosis with polyangiitis; EGPA: eosinophilic granulomatosis with polyangiitis; MPO: myeloperoxidase; P: perinuclear; PR3: proteinase 3; C: cytoplasmic; BVAS: Birmingham vasculitis activity score; FFS: five-factor score; WBC: white blood cell; PLT: platelet; ESR: erythrocyte sedimentation rate; CRP: C-reactive protein; IL: interleukin.

**Table 2:** Linear regression analysis of inflammation-related variables and serum IL-27 and IL-18 levels with the cross-sectional BVAS.

Variables	Univariable			Multivariable		
	Beta^∗^	95% CI	*P* value	Beta^∗^	95% CI	*P* value
WBC count	0.370	0.277, 1.033	0.001	-0.167	-1.172, 0.580	0.503
Neutrophil count	0.477	0.609, 1.504	<0.001	0.381	-0.166, 1.853	0.100
Lymphocyte count	-0.160	-3.845, 0.667	0.165			
PLT count	0.304	0.004, 0.027	0.007	0.124	-0.008, 0.021	0.368
Serum albumin	-0.620	-8.647, -4.748	<0.001	-0.419	-7.410, -1.635	0.003
ESR	0.388	0.037, 0.125	<0.001	0.168	-0.022, 0.092	0.225
CRP	0.359	0.027, 0.107	0.001	-0.234	-0.098, 0.010	0.112
IL-27	0.393	0.006, 0.021	<0.001	0.221	0.001, 0.015	0.025
IL-18	0.158	-0.003, 0.018	0.170			

IL: interleukin; BVAS: Birmingham vasculitis activity score; WBC: white blood cell; PLT: platelet; ESR: erythrocyte sedimentation rate; CRP: C-reactive protein. ^∗^Beta: standardised correlation coefficient.

**Table 3:** Correlation of inflammation-related variables and serum IL-18 level with serum IL-27 level.

Variables	Correlation coefficient (*r*)	*P* value
WBC count	0.037	0.748
Neutrophil count	0.120	0.300
Lymphocyte count	-0.127	0.270
PLT count	-0.056	0.629
Serum albumin	-0.384	0.001
ESR	0.166	0.148
CRP	0.210	0.066
IL-18	0.478	<0.001

IL: interleukin; WBC: white blood cell; PLT: platelet; ESR: erythrocyte sedimentation rate; CRP: C-reactive protein.

**Table 4:** Comparison of serum IL-27 and IL-18 levels according to the presence and absence of clinical manifestations.

Clinical manifestation	Serum IL-27		*P* value	Serum IL-18		*P* value
	Absence	Presence		Absence	Presence	
General manifestation	196.7 (191.4)	243.8 (251.2)	0.357	327.5 (167.5)	326.2 (151.4)	0.975
Cutaneous manifestation	213.2 (210.6)	220.1 (271.0)	0.936	323.0 (158.8)	366.9 (188.7)	0.496
Mucous and ocular manifestation^∗^	N/A	N/A	N/A	N/A	N/A	N/A
Otorhinolaryngologic manifestation	214.0 (201.1)	213.6 (233.8)	0.995	344.8 (185.0)	304.6 (122.7)	0.257
Pulmonary manifestation	214.5 (155.5)	213.3 (250.1)	0.980	325.3 (173.3)	328.2 (153.3)	0.938
Cardiovascular manifestation	214.3 (218.5)	204.8 (145.5)	0.932	326.3 (164.4)	339.8 (77.7)	0.872
Gastrointestinal manifestation^∗∗^	N/A	N/A	N/A	N/A	N/A	N/A
Renal manifestation	105.8 (163.5)	308.7 (210.8)	<0.001	270.4 (116.8)	376.7 (178.3)	0.003
Nervous systemic manifestation	222.5 (188.5)	190.6 (276.8)	0.630	337.2 (172.8)	299.8 (123.0)	0.367

Values are expressed as a mean ± standard deviation. IL: interleukin; BVAS: Birmingham vasculitis activity score; N/A: not applicable. ^∗^Since only three patients presented mucous and ocular manifestation, statistical analysis is not reliable. ^∗∗^Since only one patients presented gastrointestinal manifestation, statistical analysis is not allowed.

**Table 5:** Correlation of serum IL-27 level and serum IL-18 level with the total score of each clinical item of BVAS.

	Serum IL-27 level		Serum 18 level	
	Correlation coefficient (*r*)	*P* value	Correlation coefficient (*r*)	*P* value
General manifestation	0.084	0.468	0.011	0.924
Cutaneous manifestation	-0.062	0.590	0.043	0.707
Mucous and ocular manifestation	0.192	0.094	-0.014	0.901
Otorhinolaryngologic manifestation	0.088	0.445	-0.122	0.292
Pulmonary manifestation	0.104	0.369	0.059	0.611
Cardiovascular manifestation	-0.024	0.836	0.034	0.771
Gastrointestinal manifestation	-0.115	0.320	-0.086	0.458
Renal manifestation	0.514	<0.001	0.256	0.025
Nervous systemic manifestation	-0.002	0.987	-0.007	0.949

IL: interleukin; BVAS: Birmingham vasculitis activity score.

**Table 6:** Correlation of inflammation-related variables and serum IL-27 and IL-18 levels with BVAS according to the time of diagnosis.

Variables	Correlation coefficient (*r*)	*P* value
Newly diagnosed AAV (*N* = 40)		
WBC count	0.076	0.641
Neutrophil count	0.175	0.280
Lymphocyte count	-0.152	0.349
PLT count	0.104	0.522
Serum albumin	-0.488	0.001
ESR	0.206	0.202
CRP	0.209	0.195
IL-27	0.420	0.007
IL-18	0.431	0.005
Previously diagnosed AAV (*N* = 37)		
WBC count	0.354	0.031
Neutrophil count	0.472	0.003
Lymphocyte count	0.006	0.972
PLT count	0.174	0.304
Serum albumin	-0.248	0.140
ESR	0.200	0.235
CRP	0.296	0.076
IL-27	0.367	0.025
IL-18	-0.018	0.915

IL: interleukin; ANCA: antineutrophil cytoplasmic antibody; BVAS: Birmingham vasculitis activity score; ANCA: antineutrophil cytoplasmic antibody; AAV: ANCA-associated vasculitis; WBC: white blood cell; PLT: platelet; ESR: erythrocyte sedimentation rate; CRP: C-reactive protein.

## Data Availability

The data used to support the findings of this study are available from the corresponding author upon request.

## References

[1] Pflanz S., Timans J. C., Cheung J. (2002). IL-27, a heterodimeric cytokine composed of EBI3 and p28 protein, induces proliferation of naive CD4^+^ T cells. *Immunity*.

[2] Villarino A. V., Huang E., Hunter C. A. (2004). Understanding the pro- and anti-inflammatory properties of IL-27. *Journal of Immunology*.

[3] Charlot-Rabiega P., Bardel E., Dietrich C., Kastelein R., Devergne O. (2011). Signaling Events Involved in Interleukin 27 (IL-27)-induced Proliferation of Human Naive CD4^+^ T Cells and B Cells. *The Journal of Biological Chemistry*.

[4] Batten M., Ramamoorthi N., Kljavin N. M. (2010). IL-27 supports germinal center function by enhancing IL-21 production and the function of T follicular helper cells. *The Journal of Experimental Medicine*.

[5] Visperas A., Do J. S., Bulek K., Li X., Min B. (2014). IL-27, targeting antigen-presenting cells, promotes Th17 differentiation and colitis in mice. *Mucosal Immunology*.

[6] Murugaiyan G., Mittal A., Lopez-Diego R., Maier L. M., Anderson D. E., Weiner H. L. (2009). IL-27 is a key regulator of IL-10 and IL-17 production by human CD4+ T cells. *Journal of Immunology*.

[7] Lai X., Wang H., Cao J. (2016). Circulating IL-27 is elevated in rheumatoid arthritis patients. *Molecules*.

[8] Xia L. P., Li B. F., Shen H., Lu J. (2015). Interleukin-27 and interleukin-23 in patients with systemic lupus erythematosus: possible role in lupus nephritis. *Scandinavian Journal of Rheumatology*.

[9] Yoshizaki A., Yanaba K., Iwata Y. (2011). Elevated serum interleukin-27 levels in patients with systemic sclerosis: association with T cell, B cell and fibroblast activation. *Annals of the Rheumatic Diseases*.

[10] Jennette J. C., Falk R. J., Bacon P. A. (2013). 2012 revised International Chapel Hill Consensus Conference Nomenclature of Vasculitides. *Arthritis and Rheumatism*.

[11] Watts R., Lane S., Hanslik T. (2006). Development and validation of a consensus methodology for the classification of the ANCA-associated vasculitides and polyarteritis nodosa for epidemiological studies. *Annals of the Rheumatic Diseases*.

[12] Quartuccio L., Treppo E., Valent F., De Vita S. (2021). Healthcare and economic burden of ANCA-associated vasculitis in Italy: an integrated analysis from clinical and administrative databases. *Internal and Emergency Medicine*.

[13] Jennette J. C., Falk R. J. (2014). Pathogenesis of antineutrophil cytoplasmic autoantibody-mediated disease. *Nature Reviews Rheumatology*.

[14] Lamprecht P., Kerstein A., Klapa S. (2018). Pathogenetic and clinical aspects of anti-neutrophil cytoplasmic autoantibody-associated vasculitides. *Frontiers in Immunology*.

[15] Xu Y., Xu H., Zhen Y. (2019). Imbalance of Circulatory T Follicular Helper and T Follicular Regulatory Cells in Patients with ANCA-Associated Vasculitis. *Mediators of Inflammation*.

[16] Zhao Y., Lutalo P. M., Thomas J. E. (2014). Circulating T follicular helper cell and regulatory T cell frequencies are influenced by B cell depletion in patients with granulomatosis with polyangiitis. *Rheumatology*.

[17] Abdulahad W. H., Lepse N., Stegeman C. A. (2013). Increased frequency of circulating IL-21 producing Th-cells in patients with granulomatosis with polyangiitis (GPA). *Arthritis Research & Therapy*.

[18] Yoon T., Ahn S. S., Song J. J., Park Y. B., Lee S. W. (2019). Serum interleukin-21 positivity could indicate the current activity of antineutrophil cytoplasmic antibody-associated vasculitis: a monocentric prospective study. *Clinical Rheumatology*.

[19] Shen H., Xia L. P., Lu J. (2013). Elevated levels of interleukin-27 and effect on production of interferon-*γ* and interleukin-17 in patients with Behçet's disease. *Scandinavian Journal of Rheumatology*.

[20] Mukhtyar C., Lee R., Brown D. (2009). Modification and validation of the Birmingham Vasculitis Activity Score (version 3). *Annals of the Rheumatic Diseases*.

[21] Guillevin L., Pagnoux C., Seror R. (2011). The five-factor score revisited: assessment of prognoses of systemic necrotizing vasculitides based on the French Vasculitis Study Group (FVSG) cohort. *Medicine*.

[22] Bossuyt X., Cohen Tervaert J. W., Arimura Y. (2017). Revised 2017 international consensus on testing of ANCAs in granulomatosis with polyangiitis and microscopic polyangiitis. *Nature Reviews Rheumatology*.

[23] McAdoo S. P., Medjeral-Thomas N., Gopaluni S. (2019). Long-term follow-up of a combined rituximab and cyclophosphamide regimen in renal anti-neutrophil cytoplasm antibody-associated vasculitis. *Nephrology, Dialysis, Transplantation*.

[24] Hunter C. A., Kastelein R. (2012). Interleukin-27: balancing protective and pathological immunity. *Immunity*.

[25] Wittmann M., Doble R., Bachmann M., Pfeilschifter J., Werfel T., Mühl H. (2012). IL-27 regulates IL-18 binding protein in skin resident cells. *PLoS One*.

[26] Choi Y. H., Lim E. J., Kim S. W., Moon Y. W., Park K. S., An H. J. (2019). IL-27 enhances IL-15/IL-18-mediated activation of human natural killer cells. *Journal for Immunotherapy of Cancer*.

[27] Pressler B. M., Falk R. J., Preston C. A. (2006). Interleukin-18, neutrophils, and ANCA. *Kidney International*.

[28] Hewins P., Morgan M. D., Holden N. (2006). IL-18 is upregulated in the kidney and primes neutrophil responsiveness in ANCA-associated vasculitis. *Kidney International*.

[29] Flossmann O., Bacon P., de Groot K. (2008). Development of comprehensive disease assessment in systemic vasculitis. *Annals of the Rheumatic Diseases*.

